# A Social–Ecological Study of Perceptions and Determinants of Sexual Enhancement Drug Use among Men and Women in Ghana

**DOI:** 10.3390/ijerph19116521

**Published:** 2022-05-27

**Authors:** Padmore Adusei Amoah, Stephen Baffour Adjei, Francis Arthur-Holmes

**Affiliations:** 1 School of Graduate Studies, Lingnan University, Tuen Mun, Hong Kong; 2 Institute of Policy Studies, Lingnan University, Tuen Mun, Hong Kong; 3 Department of Applied Psychology, Lingnan University, Tuen Mun, Hong Kong; 4 Department of Interdisciplinary Studies, Akenten Appiah-Menka University of Skills Training and Entrepreneurial Development, Kumasi P.O. Box 1277, Ghana; stevoo24@yahoo.com; 5 Department of Sociology and Social Policy, Lingnan University, Tuen Mun, Hong Kong; frarthur88@gmail.com

**Keywords:** aphrodisiac, sexual enhancement drugs, gender, health literacy, public perceptions, Ghana

## Abstract

The use and sale of sexual enhancement drugs (particularly unapproved aphrodisiacs) have become a public health concern in Ghana and many other sub-Saharan African countries. While most studies have examined this phenomenon from the level of individual perspectives, this study investigates the multi-dimensional and multi-level factors (e.g., individual characteristics and behaviours, interpersonal factors, community norms and practices, institutional and public policy factors) that influence attitudes, perceptions, and use of aphrodisiacs among men and women in Ghana. Using a concurrent mixed-method design, we derived the data from a semi-structured interview and cross-sectional survey conducted across five administrative regions in Ghana. Interpretative phenomenological analysis and logistic regression techniques were used to analyse the qualitative and quantitative (survey) data, respectively. Approximately 12.6% of participants (17.6% among males and 7.2% among females) had used an aphrodisiac in the six months prior to the study. Approximately 23.4% of the participants had more than one partner during the same period. Among men, being religious (B = −0.238, *p* < 0.05) and having multiple sexual partners (B = 0.481, *p* < 0.01) were positively associated with the use of aphrodisiacs. For women, being employed (B = −1.539, *p* < 0.01), engaging in physical activities (exercising) (B = −0.658, *p* < 0.05), having good health (B = 0.869, *p* < 0.05), having multiple sexual partners (B = 1.191, *p* < 0.01), and taking alcohol (B = 1.041, *p* < 0.01) were associated with use of aphrodisiacs. Although many participants had used aphrodisiacs, women, in particular, held unfavourable views about the drugs due to perceived negative health implications for themselves and their partners. The findings also show that community-level factors (e.g., social norms and expectations), interpersonal factors (e.g., expectations of partners and friends), public policy (e.g., drug-related regulations), and organisational/institutional factors (e.g., health system arrangements about access and use of drugs) were critical to the sale and use of aphrodisiacs among both men and women in Ghana. A multi-level analysis of the use of sexual enhancement drugs among men and women is crucial to formulating social and public health policies that aim to improve public knowledge of these drugs, reduce uncontrolled production, and protect population health and well-being.

## 1. Introduction

Research reports show that an increase in unfulfilled sexual desires and sexual dysfunction globally has led to a rise in the prevalence and use of approved and unapproved aphrodisiacs/sexual enhancement drugs in many places [1,2]. Although aphrodisiacs, i.e., any food or drug that enhances sexual arousal, desire, pleasure, and performance [3,4,5], have been historically used for recreational and reproductive health purposes, public health concerns have prompted an increase in research on the subject across sub-Saharan Africa (SSA), particularly in the last decade [4,6]. Aphrodisiacs are thought to support overall sexual health and well-being, stimulating sexual arousal and performance with the support of pharmacological compounds and a perceived psychological boost in sexual desire [2,3,5]. As in many other sub-Saharan African countries, the commonly used sexual enhancement drugs that are approved by the national regulatory body in Ghana (i.e., Food and Drug Authority, FDA) often take the form of alcoholic/non-alcoholic drinks (e.g., Adonko bitters and Alomo bitters) and pills/capsules (e.g., Angel Natural Capsule, Kingdom Ginseng Power Capsule) usually made from herbal ingredients. Unapproved substances include alcoholic and non-alcoholic mixtures, with the main ingredients being herbs such as mahogany, ginger, aidan fruit, and West African black pepper [2,7,8]. Other users mix acholic substances with drugs such as paracetamol and tramadol. Unregistered drug hawkers also sell dried or partly dried tree barks, flowers, and leaves that can be consumed directly or used for concoctions at home [2,9].

The reasons for the use of aphrodisiacs among men and women tend to be similar across SSA. These reasons include the desire to prolong sexual intercourse, increase sexual desire and satisfaction, and enhance aggression during sexual intercourse [7,10,11]. Among men, such reasons include addressing erectile dysfunction and premature ejaculation [12] and enhancing self-confidence and adherence to embedded sexual scripts of masculinity [11]. However, continuous and uncontrolled use of these drugs can cause erectile dysfunction, dizziness, seizures, headache [9,13], painful intercourse, and vaginal dryness and discharge [10,11]. These drugs are also associated with the risk of infection from sexually transmitted diseases (e.g., HIV), as well as chronic diseases such as liver diseases and renal dysfunction [6,14,15,16,17]. Moreover, aphrodisiac use increases the exchange of more body fluids because of prolonged sexual activity, which increases the chance of contracting sexually transmitted diseases [17]. Alcohol-based aphrodisiacs can encourage risky practices such as unprotected sex (e.g., not using a condom), which increases sexually transmitted infections [16].

Despite the increasing demand for and use of aphrodisiacs and their potential undesirable effects, public knowledge of using such drugs remains understudied and thus requires further interrogation [9]. Recently, there has been an interest in the gender dimension of aphrodisiac use in SSA because of increased female usage [2,18]. For instance, some studies report that 42–53.9% of women use various kinds of herbal medicines, including aphrodisiacs, in parts of Nigeria [10,18], while 61% of men and 46% of women have been reported to use aphrodisiacs in Ghana [12]. These gendered differences require more investigation to formulate policies that address practical needs and purposes.

Most studies in Ghana have explored the prevalence of aphrodisiacs, the influence of masculinity on the use of aphrodisiacs, and women’s experience of sexual pleasure, either from quantitative or qualitative perspectives [2,7,13,19,20]. However, differences in the correlates of aphrodisiac use among men and women have not been adequately explored in the extant research in Ghana. Additionally, a multi-level analysis (i.e., taking into consideration individual and societal factors) that focuses on matters of sexual health knowledge, public perception, and underlying reasons for the use of such drugs is missing in the existing literature. This study employs a concurrent mixed-method design to examine public perceptions of the prevalence, correlates, and implications of aphrodisiac use among men and women to fill this important knowledge gap in Ghana and other contexts. The study explores the multi-level characteristics and behavioural correlates of aphrodisiac use among men and women. The study addresses the following research questions: (1) What are the socio-demographic and behavioural factors associated with aphrodisiac use among men and women in Ghana? (2) Why do men and women use aphrodisiacs within the socio-cultural milieu of Ghana? and (3) How do micro and macro factors affect public perceptions, attitudes, and knowledge of aphrodisiac use in Ghana?

While this study enriches existing knowledge about aphrodisiac use, it also contributes to the ongoing fight against the proliferation and use of unapproved recreational drugs and risky sexual practices by providing insight into public attitudes and gendered perspectives on aphrodisiacs [1,13]. Considering the growing double burden of disease in many low- and middle-income countries, including Ghana, studies of this nature are crucial to protecting the already weak but stretched health and social policy systems in such places [21,22]. This study focuses on heterosexual relationships, which are the commonest and sanctioned by cultural and religious doctrines as well as legal stipulations in Ghana [2,23,24].

### Aphrodisiac Use among Men and Women: A Multi-Level Theoretical Perspective

To understand the dynamics of the prevalence and use of aphrodisiacs in Ghana beyond individual characteristics and behaviours, the study uses the social–ecological model of health and health behaviours [25,26] as a relevant theoretical framework. The social–ecological model argues that the health outcomes and related behaviours of individuals are shaped by a constellation of five hierarchical factors lying at the micro and macro levels of societies: they relate to (1) intrapersonal and (2) interpersonal factors; (3) the beliefs and social norms of a community; (4) organisational factors, and (5) public policy as a whole [25,26]. The intrapersonal factors describe individual demographic characteristics (e.g., age, sex, and educational attainment), beliefs, knowledge, and attitudes about health and related behaviours. For instance, while men use aphrodisiacs to prolong erection, women tend to use them for vaginal cleansing and to enhance the sexual pleasure of their partners [11,27].

Aside from these intrapersonal elements, forces outside individual beliefs and characteristics influence behaviours and decisions about aphrodisiac use. From the social–ecological model, one of them is interpersonal relationships, which deals with how social relationships affect the perception of health problems and decisions about how to address such problems and behaviours on specific treatments and drugs [25]. People may use aphrodisiacs to appease the pressures placed on them by their partners or friends [13]. The perception, attitudes, and utilisation of aphrodisiacs are also conditioned by cultural norms, expectations, values, and societal and religious beliefs in a given community (i.e., geographic area or a group) [28]. These norms, expectations, and acceptable behaviours shape individual beliefs and the function and formation of social relationships [25]. In many sub-Saharan African settings, such community structures strongly dictate sexual relations, expectations, and perceptions in heterosexual relationships, such as promoting hegemonic masculine ideals [2,28].

The organisational aspect of the social–ecological model describes the characteristics and operations of social institutions and their role in population health and well-being [25]. It includes health system structures and arrangements at local and national levels that deal with health promotion activities geared towards behavioural change. The organisational aspect also includes communication between the different divisions of a health system, and between categories of health professionals about their experiences [25,26,29]. The purpose of these structures and institutions is to develop the health knowledge of a population and to cultivate organisational cultures that promote good health [29]. Another factor that explains the prevalence of aphrodisiac use relates to public policy and regulations governing the production, commercialisation, and utilisation of such products [28]. Public policies determine the extent to which such products are manufactured and made available to the public. Public policy also comprises the laws governing sexual practices [28]. For example, the commercialisation of aphrodisiacs is partly due to provisions made by public policy. Thus, the social–ecological model offers individual and macro-level perspectives on the prevalence and perceptions of aphrodisiac use among men and women in Ghana, and the implications of these behaviours and practices for the health system and related social policies.

## 2. Methods

### 2.1. Study Design and Sampling

This study is part of a concurrent mixed-method cross-sectional study that examines the social aspects of health and well-being among young and adult Ghanaians. This study draws data from the larger research work conducted in 2018 across five of the then ten administrative regions of Ghana using a multi-cluster sampling approach. The five regions (Brong Ahafo, Greater Accra, Ashanti, Eastern, and Northern) and their communities were selected purposively to ensure geographical and socio-economic balance across the country. Likewise, 29 districts and 128 communities (51 rural and 77 urban areas) across the five regions were purposively selected due to their relevance to this study. The inclusion criteria comprised religious and ethnic composition, geographical balance, sex, and socio-economic characteristics of residents in the areas, which were obtained with the help of the 2012 population and housing data and living standard survey reports [30,31]. A systematic sampling technique was employed to select a person consenting to participate in the study in every second and fifth house in rural and urban areas, respectively. The study used a house as a unit for selecting participants because previous experiences indicate that people in the same houses often share similar socio-economic characteristics. This approach was adopted to facilitate the capture of a sample with as much diversity as possible. Overall, the broader study comprised 2097 survey participants. The objective was to obtain a minimum of 384 participants for each of the five regions, as deduced from sampling criteria identified elsewhere [32]. This study analysed the responses of 1929 participants. Detailed descriptions of the broader study’s sampling process have been reported elsewhere [33].

However, the qualitative data were gathered from four regions: the Ashanti, Brong Ahafo, and Greater Accra regions. An interpretive phenomenological approach [34] was used in this study in recognition of the fact that perceptions, experiences, and evaluation of aphrodisiac use and other sexual behaviours in Ghana are embedded in the sociohistorical and inter-subjective understanding and experiences of people [2,34,35]. This approach is also consistent with the tenets of the social–ecological model employed for this study. Participants were purposively selected to have a balance in terms of sex (i.e., men and women), age, religiosity and religious affiliation, ethnicity, marital status, sexual activity, and geographical composition (rural and urban residents). Participants were recruited from their homes, workplaces, and other public places. Semi-structured interviews were conducted with 31 participants. Nine participants took part in the qualitative study and the survey. The interviews took place at a location preferred by the participants. This was to ensure that people had the privacy to express their views and experiences, considering that sexual relations and related subjects have strict cultural and religious interpretations and are considered “immoral”, particularly among unmarried persons in Ghana [20]. The interview topics covered issues at the micro and macro levels of Ghanaian society, such as participants’ beliefs about and awareness of aphrodisiacs, their perceptions and attitudes about the efficacy and effects (e.g., health) of the drugs, the role of relevant institutions, and the reasons that people use them. These topics were discussed at both the micro- and public policy levels, and in terms of the reasons for the rise in utilisation. The interviews were predominantly conducted in Twi and English, depending on participants’ fluency. Each interview lasted around 45 minutes and was audio-recorded, with the consent of participants. 

### 2.2. Measures

#### 2.2.1. Dependent Variable: Use of Aphrodisiacs

Participants were asked to respond to the statement “I have taken aphrodisiacs prior to or during sexual encounters in the past six months” on a four-point Likert scale ranging from “never” to “always”, with the fifth option being “not applicable”. Trained interviewers explained the question with specific examples to participants. Either conventional or non-conventional forms of aphrodisiacs were emphasised.

#### 2.2.2. Independent Variables

These variables comprised primarily intrapersonal factors such as age (in years), monthly income/stipend, subjective socio-economic status (rated from 1 = low to 10 = high), and religiosity, which measures “one’s pious conformity to a religion through practice and conduct (e.g., how often one goes to church/mosque)” [28]. Participants were asked to indicate how religious they were on a seven-point scale, ranging from “extremely non-religious” to “extremely religious”. Others included educational attainment (“never been to school” to tertiary education), and employment status (employed, retired, unemployed). Behavioural factors, which comprised the frequency of physical activities (exercising), smoking, and alcohol intake, were also measured. These were measured on a five-point Likert scale ranging from “never” to “daily”. We also measured sexual behaviours such as the number of sexual partners in the past six months and whether a person (or their partner) used a condom the last time they had sex (yes/no). Their overall health status was measured by asking participants to rate their health on a five-point scale, from “poor” to “excellent”.

#### 2.2.3. Covariates

The covariates included region of residence, rural or urban residence, marital status (married, divorced, widowed, separated, single/never married, and living together as married). and whether a person had had sexual intercourse in the past six months (yes/no).

### 2.3. Data Analyses

In order to provide an in-depth perspective on the research topic, a side-by-side technique was employed for the data analyses. By this approach, the results of the quantitative and qualitative aspects of the data were presented separately but interpreted together [36].

#### 2.3.1. Quantitative Analysis

The quantitative analysis comprised descriptive analysis that examined the characteristics of the data, as shown in Table 1. As the data for the dependent variable were not normally distributed (Kolmogorov–Smirnov, B = 0.508, *p* < 0.000), Spearman’s rank correlation analyses were conducted to include only independent variables that correlated with the dependent variable in the regression analyses. However, marital status was included in the regression analyses regardless of its correlation with the dependent variable. This was because sexual relations in the Ghanaian society are culturally considered as the preserve of married couples [2,20]. The inferential analysis used the ordinal logistic regression technique to identify personal and behavioural characteristics associated with aphrodisiac use. The analyses were split between men and women. Cases with missing or inapplicable responses were left unchanged. Significant relations were set at *p* < 0.05.

#### 2.3.2. Qualitative Analysis

We analysed the data based on insights drawn from interpretative phenomenological analysis (IPA) [34,37]. The IPA allowed the emerging themes from the interviews and their connection to the social–ecological model to be developed [38]. Thus, given the use of the social–ecological model in this study, an inductive analysis approach was applied, although flexibility was allowed to consider other emerging perspectives. The process started with open coding by two authors to identify categories of observations for each interview. The analysis then focused on the meanings that participants made of their personal and social worlds. Each author then converted the categories into emerging themes from the interviews. The two authors co-developed the emerging themes into superordinate themes, which formed initial findings. A third analyst independently reviewed the analysis process and the superordinate themes generated. Feedback from the third analyst was then integrated into the analysis for the resultant themes reported as findings in this study.

## 3. Results

### 3.1. Quantitative Study

Table 1 presents the descriptive statistics of the variables in the study according to sex. The average age of participants was 38 years, with 50.7% of them being males. Most of the participants (23.7%) had completed middle school/junior high school. Approximately 40% of them were married at the time of the survey. Regarding their health and related behaviours, the majority of the participants had never consumed alcoholic drinks (77.2%) or smoked (84.5%). Most participants rated their health as “good” (39.1%), but a significant proportion also described their health as either “fair” or “poor” (24%). Around 49.9% (males = 53.6%; females = 45.9%) of participants had had sexual intercourse within the six months prior to the survey, with many (23.4%) of them having been with more than one partner during the same period. Around 12.6% of them (17.6% of males and 7.2% of females) had used aphrodisiacs during the six months prior to the study. There were significant differences between the men and women in relation to income, educational status, employment status, and marital status. The females were less likely to consume alcohol, smoke, have multiple sexual partners, have unprotected sex, and use aphrodisiacs. The males were more likely to perform physical activities (exercising) and describe their health in positive terms.

**Table 1:** Descriptive statistics of variables in the study.

	Men		Women		*p*-Value	Overall	
Variable	Mean/*n* (*n* = 978)	SD/%	Mean/Valid *n* (*n* = 951)	SD/%		Mean/Valid *n* (*n* = 1929)	SD/%
**Age (in years): Mean/Range**	38.43/18–85	16.7	37.72/18–91	16.28	0.339	38.08/18–91	16.49
**Monthly Income (** **GHS)/Range**					**0.012** ^a^		
	590.02/7–6500	732.17	539.92/7–671			568.33/7–6500	706.4
**Socioeconomic Status (SES)**	4.07	1.94	3.94	1.75	0.139 ^a^	4.01	1.85
**Region of residence**					**0.000**		
Ashanti	213	21.8	245	25.8		458	23.7
Greater Accra	185	18.9	187	19.7		373	19.3
Brong Ahafo	164	16.8	192	20.2		356	18.4
Northern Region	274	28.0	157	16.5		431	22.3
Eastern Region	142	14.5	170	17.9		312	16.2
**Area of residence**					**0.038**		
Rural	345	35.3	379	39.9		724	37.5
Urban	633	64.7	572	60.1		1205	62.5
**Educational attainment**					**0.000**		
Never been to school	160	16.4	203	21.4		365	18.9
Primary school	149	15.2	175	18.4		324	16.8
Middle School/JHS/O level	242	24.7	216	22.7		457	23.7
Secondary School/A level	219	22.4	192	20.2		411	21.3
Tertiary (including postgraduate)	208	21.3	165	17.3		372	19.3
**Employment status**					**0.000**		
Employed	596	61.4	558	59.3		1155	60.4
Pension/Retired	47	4.8	23	2.4		70	3.7
Unemployed	328	33.8	360	38.2		688	36.0
**Marital status**					**0.034**		
Married	389	39.8	386	40.6		775	40.2
Divorced	20	2.0	10	1.1		30	1.6
Widowed	28	2.9	45	4.7		73	3.8
Separated	12	1.2	22	2.3		34	1.8
Single (Never married)	495	50.6	462	48.6		957	49.6
Living together as married	34	3.5	26	2.7		60	3.1
**Religiosity**	5.67	1.11	5.78	0.92	0.087 ^a^	5.71	1.02
Minimum-maximum						1–7	
**Alcohol intake**					**0.000** ^a^		
Never	658	70.9	765	83.5		1424	77.2
Once a month or less often	130	14.0	90	9.8		220	11.9
Several times a month	74	8.0	28	3.1		102	5.5
Several times a week	29	3.1	15	1.6		44	2.4
Daily	37	4.0	18	2.0		55	3.0
**Physical activities**					**0.000** ^a^		
Never	201	21.8	262	28.6		463	25.2
Once a month or less often	159	17.3	194	21.2		353	19.2
Several times a month	238	25.8	197	21.5		435	23.7
Several times a week	171	18.6	155	16.9		327	17.8
Daily	152	16.5	108	11.8		260	14.1
**Smoking**					**0.000** ^a^		
Never	719	78.1	829	91.0		1549	84.5
Once a month or less often	40	4.3	20	2.2		60	3.3
Several times a month	83	9.0	31	3.4		114	6.2
Several times a week	34	3.7	17	1.9		51	2.8
Daily	45	4.9	14	1.5		59	3.2
**Health status**					**0.009** ^a^		
Poor	40	4.2	58	6.2		98	5.2
Fair	169	17.9	185	19.8		354	18.8
Good	370	39.2	364	39.0		735	39.1
Very good	237	25.1	227	24.3		464	24.7
Excellent	128	13.6	99	10.6		227	12.1
**Sexual intercourse**					**0.001**		
No	437	46.4	491	54.1		928	50.1
Yes	505	53.6	417	45.9		922	49.8
**Number of sexual partners**					**0.000** ^a^		
0	71	7.3	60	6.3		131	10.9
1	343	35.1	444	46.7		788	65.7
2	144	14.7	30	3.2		174	14.5
3	64	6.5	13	1.4		77	6.4
4	14	1.4	5	0.5		19	1.6
5	2	0.2	6	0.6		2	0.2
6	1	0.1	--	--		7	0.6
8	1	0.1	--	--		1	0.1
*Mean/SD*	*1.41*	*0.965*	*1.07*	*0.774*		*1.25*	*0.897*
**Use of condom**					**0.004**		
No	378	38.7	423	44.5		801	41.6
Yes	201	20.6	156	16.4		357	18.5
**Use of aphrodisiacs**					**0.000** ^a^		
Never	548	82.4	586	92.7		1134	87.4
Sometimes	93	14.0	35	5.5		128	9.9
Most of the times	18	2.7	7	1.1		25	1.9
Always	6	0.9	4	0.6		10	0.8

Note: Some values may not add to the total sample due to missing or inapplicable responses. JHS = Junior High School. *p*-value: Bold figures indicate *p* < 0.05; --: No response recorded; ^a^ *p*-value based on independent sample *t*-test. All other *p*-values are based on Chi-Square tests.

According to Table 2, among men, being religious (B = −0.238, *p* < 0.05) was negatively associated with aphrodisiac use, while having multiple sexual partners (B = 0.481, *p* < 0.01) was positively associated with the use of aphrodisiacs. Among women, being employed (B = −1.539, *p* < 0.01) and engaging in physical activities (exercising) (B = −0.658, *p* < 0.05) were negatively associated with the use of aphrodisiacs. However, having good health (B = 0.869, *p* < 0.05), multiple sexual partners (B = 1.191, *p* < 0.01), and consuming alcohol (B = 1.041, *p* < 0.01) were positively associated with aphrodisiac use among females. In the overall sample, being a man (B = 1.343, *p* < 0.001) and having multiple sexual partners (B = 0.757, *p* < 0.001) were associated with the use of aphrodisiacs, and being religious (B = −0.269, *p* < 0.01) was negatively correlated with aphrodisiac use.

### 3.2. Qualitative Study

Participants were mostly aged between 18 and 35 years (45.2%), and most of them were males. Most of them had completed junior high school (45.2%). Approximately 54.8% of them were not married, as shown in Table 3.

The qualitative part of the study yielded five inter-related themes: prevalence of aphrodisiacs, reasons for the increasing use of aphrodisiacs, perception and attitudes towards aphrodisiacs, concerns about the effect of aphrodisiacs on health, and knowledge of safe sexual relations. These themes underscore the increasing use of aphrodisiacs. The themes were linked to specific social–ecological factors, such as intrapersonal, interpersonal, community, and public policy factors.

### 3.3. Prevalence of Aphrodisiacs

Participants reported that aphrodisiacs, especially unapproved ones, had become a prominent feature of the local sexual relations landscape. Primarily, they attributed the prevalence of the drugs, both those on the market and even homemade ones, to weaknesses in public regulatory mechanisms. The drugs were so common that some parents were worried about their children taking such drugs without proper guidance. As one female interviewee indicated, “I am worried about it. Our children will go for those drugs all because of the adverts” (Interview 12, Female).

Both approved and unapproved drugs from orthodox and traditional sources were available. The unapproved drugs often comprised homemade concoctions, mainly from herbs, as indicated by a male participant: “For me, I have a drug I take; not the ones they advertise…herbs…I prepare by myself” (Interview 15, Male). Unapproved drugs also included imported drugs, especially those from China, which had become popular among young men and women, mainly to increase their libido and prolong climax during sexual intercourse. One male participant averred that “The majority of the drugs are Chinese drugs... if you take the drug, you can have sex with a woman till the next day, and the penis will still be erect” (Interview 11, Male).

To the participants, the unapproved drugs were imported illegally. They attributed the proliferation of unapproved drugs to the poor enforcement of food and drug regulations and inadequate employment opportunities. These factors encourage individuals and companies to produce and market these drugs illegally. As one male participant asserted, “In Ghana, you can sell and buy any drug outside, but it is not the same in Cote d’Ivoire” (Interview 16, Male). This view was corroborated by a female participant: “The security at our borders is weak …. even if border security isn’t tight, can’t we arrest those people after the drugs are in the system?” (Interview 12, Female).

### 3.4. Reasons for the Increasing Use of Aphrodisiac

Participants mostly tied the reasons associated with the use of aphrodisiacs to intrapersonal, interpersonal, and community factors. Regarding the intrapersonal factor, a common reason for the rising use of aphrodisiacs and other erotic drugs was perceived as poor sexual performance and sexual dissatisfaction among both sexes, especially men. For the intrapersonal and community factors, poor performance was associated with poor diets as part of changing societal behaviours about food and nutrition, which have rendered many people unhealthy and sexually weak. For example, one female participant indicated that “People are sick [sexually weak]. People cannot have sex with their partners, so they take drugs…” (Interview 1, Female). The use of these drugs among women was often to meet the expectations of their partners. To some extent, the participants’ views pointed to a social practice where women were expected or even obliged to satisfy the sexual needs of their partners. Women often use drugs to boost their libido and satisfaction and make their private parts extra sensual for their partners. The women felt that consuming these drugs was important to please and show gratitude to their partners. For example, a male participant said, “It’s the same because some women are weak [sexually]. They have no sexual feelings, so those drugs enhance their desire.…” (Interview 13, Male). Another male participant supported this view by saying that “Some women take the drugs just to impress their men. …Maybe a man has bought a woman a car or built a house for her …” (Interview 15, Male).

Both men and women also attributed the use of aphrodisiacs to the cultural and social expectations that men must be strong in bed—lasting longer during sex and having above-average manhood size. Hence, interpersonal reasons such as avoiding ridicule by the opposite sex or peers encouraged some people to take aphrodisiacs to address these specific personal needs. As illustrated by a female participant, “Some men take drugs to increase the size of their manhood” (Interview 10, Female). A male participant also supported this view, “A man has to be strong. it’s a disgrace if you’re sexually weak” (Interview 13, Male). Consistent with the need to avoid disgrace and ridicule because of poor sexual performance, some participants felt that the urge to take the aphrodisiacs was because of sheer “wickedness”—having a “punitive” or “revengeful” mindset —among some men. As indicated by some female participants, “Some of the men are wicked. They believe that if their ladies show pain during sex, then they are ‘men’” (Interview 8, Female). Another also suggested that “Some men spend money on girls without being able to have sex with them, so when the girl becomes available, they take the drugs to have their money’s worth” (Interview 5, Female). The above reasons resonate with other facets of community factors. Some participants attributed the rise in aphrodisiac use to decaying societal morals—disregard for social and behavioural norms. They claimed that promiscuity had become a lifestyle in the Ghanaian society, which was one of the underlying factors for aphrodisiac use: “I think fornication and adultery have become rampant. Women want to try different men … so the men fear losing their women. Because of this, the men go for those drugs” (Interview 10, Female). 

To many participants, the decaying morals were also connected to a rise in advertisements about aphrodisiacs. They intimated that advertisements and peer pressure underpinned the desire of many people to try aphrodisiacs regardless of their condition, as exemplified in the following extract: “Previously, aphrodisiacs were not advertised; people just knew about them. But now, there are adverts on radios and televisions everyday…even if a person is fine [sexually], they’ll be tempted to try because of the adverts…” (Interview 3, Female). A male participant added, “Some also take advice from friends when they [the friends] recommend the drugs to them” (Interview 11, Male).

### 3.5. Perception and Attitudes towards Aphrodisiacs

Perceptions and attitudes towards the use of aphrodisiacs differed depending on the gender, marital status, and age of participants. Thus, intrapersonal factors were the primary reasons for people’s attitudes and perceptions. Two groups were identified: one having a more favourable attitude towards such drugs and therapies, while another vehemently opposed them. In general, women, and some men, did not have many favourable perceptions of aphrodisiacs. Some women felt that these drugs were unnecessary for sexual relations as they brought pain instead of pleasure to women. Some of them were even willing to divorce/reject their partners who used aphrodisiacs. For example, one female participant said, “If I see [that a partner is using aphrodisiacs], I’ll leave him the same day…” (Interview 2, Female). A male participant supported this view: “I won’t take it [even if my partner requests aphrodisiacs]. It’ll destroy my sex life in the future…she can leave; I came to this world alone” (Interview 6, Male).

Some also disliked aphrodisiacs due to their momentary effect and the economic implications of indulging in them. In one interview, a female participant rhetorically said, “That means you have to take drugs whenever you have sex. So, what happens if you don’t have money for the drugs?” (Interview 4, Female). Even those who held positive attitudes toward aphrodisiacs were concerned about the abnormal physical effects of the drugs. Hence, they were cautious about using aphrodisiacs themselves or encouraging their partners to do so, especially the unapproved ones. At worst, some women suggested that they would instead encourage their partners to see a qualified doctor for help. Some women also believed that the only condition that would make them maintain a relationship with a sexually weak man was if they were married. Otherwise, they were willing to end their relationships. For example, one female participant stated, “…if you last seconds during sex, I will tell you to get a drug but not the one that rapidly increases your performance. … It’s better to see a doctor” (Interview 5, Female). Another added, “…if you’re not my husband, I’ll leave [if a partner is not ready to take aphrodisiacs] because nobody likes a non-performing man” (Interview 21, Female). With regard to men, some perceived it as a sign of affection if their partners suggested aphrodisiacs to them to overcome their sexual weakness: “If she doesn’t love you, she won’t tell you to seek help…. It may be because of something else she wants from you, but a lady who loves you will tell you” (Interview 15, Male).

In addition to the intrapersonal factors, community norms, beliefs, practices, and expectations about sexual behaviours and sexual relationships shaped participants’ perceptions and attitudes about aphrodisiacs and all sex-related activities. The participants had conservative attitudes towards sexual acts and topics, even among married people. Some married participants felt that aphrodisiacs were the preserve of single people and expressed abhorrence towards the use of sex enhancement drugs: “All these [use of aphrodisiacs] are done by unmarried men. Married men would never use such a stupid drug” (Interview 2, Female). Additionally, the conservative socio-cultural norms meant that women were hesitant to discuss issues about sexual enhancement drugs with their partners: “…some women think they’ll be divorced/jilted if they discuss sexual performance and those drugs with their partners. They are afraid of being tagged as promiscuous by their spouses” (Interview 12, Female).

### 3.6. Concerns about the Effect of Aphrodisiacs on Health

Participants’ attitudes towards aphrodisiacs were associated with perceptions about what the use of the drugs would do to their health, especially unapproved drugs. Both men and women expressed concerns about the potential long-term effects of aphrodisiacs, including abnormal erection, consequential illness, and even death. As one female participant narrated a friend’s story, “A friend used to take certain tablets before having sex. He’s only 46 years, but he has stroke now. …Another person took tramadol to have sex, and he became very weak …he died later at a hospital” (Interview 8, Female). 

In their view, the main reason that many people continue to use unapproved substances is a misunderstanding of the potential side effects of some drugs, as well as impatience with periods of low sex drive and performance (we elaborate more on this issue under the next theme): “Some women don’t know that those drugs have side effects; that is why they push their partners to take them. They also have to be patient and understand that a man can’t perform well every time…” (Interview 14, Male).

Given these misunderstandings and impatience, some took all kinds of aphrodisiacs regardless of the health implications because they feared being divorced/rejected by their partners. One male participant also shared his friend’s ordeal: “A friend of mine takes it, and his penis will still be erect even after the sex. He says the girl likes it…” (Interview 14, Male). The health concerns also emerged from the misuse of drugs approved for purposes other than improving sexual performance and satisfaction: “Like tramadol…. It has its purpose, but some men buy it for sexual performance” (Interview 23, Female). Moreover, participants who had reservations about the stigma and health implications of aphrodisiacs preferred to use herbal or homemade remedies instead. Such remedies were perceived as safer compared to unapproved imported therapies: “I think the herbal ones are better. The foreign drugs can reduce the size of your penis” (Interview 11, Male). However, there were indications that even the revered herbal drugs, including homemade ones, could have severe adverse effects, as some participants attested: “I know a herbal medicine that someone took and had an erection for about three hours” (Interview 10, Female).

### 3.7. Knowledge of Safe Sexual Relations Underlies the Increasing Use of Aphrodisiacs

Another intrapersonal factor relating to the use of aphrodisiacs had to do with the public’s knowledge, skills, and experience when it comes to such drugs. Some participants felt that the health consequences that people suffered by taking some sexual enhancement drugs were often due to limited knowledge of the substances, particularly regarding the dosage and side effects. For instance, one female participant indicated, “I know about three people who were taken to the hospital after taking tramadol to have sex. Some of them had no idea of what it was when they took it… they only knew it makes them perform better” (Interview 3, Female).

The attitudes and stigma attached to certain sexual relations (e.g., as a sinful and private subject) compelled females, in particular, to be less curious and forthcoming about their sexual desires and preferences, leading to low knowledge of safe sex and sexual needs. As presented earlier, some participants claimed that being open about their sexual desires was considered promiscuous, which discouraged even males from learning about sexual issues, including aphrodisiacs. Apart from the socio-cultural dimensions, participants felt that people indulged in unapproved and unprescribed aphrodisiacs mainly because they had less regard for their health as they prioritised pleasure over healthy lives: “Some people want instant satisfaction, not what will happen to them in the future” (Interview 10, Female). The participants widely acknowledged that low sex education was a major reason for the uptake of “needless” drugs. They alluded to limited knowledge among both men and women on ways to satisfy their partners without the use of drugs as the reason for which people indulge in aphrodisiacs: “…the number of ‘rounds’ [sexual intercourse] doesn’t matter … But many men don’t know this, so they use drugs…” (Interview 6, Male). This was supported by a female participant, who shared that “Many women apply vaginal creams and other drugs because they don’t know how to prepare themselves for sex. …Many men don’t know that a woman’s orgasm is different from a man’s… It all boils down to education” (Interview 10, Female).

## 4. Discussion

This study has examined the correlates, perceptions, and implications of aphrodisiac use among men and women in Ghana. The study argues that the prevalence, utilisation, and perceptions about aphrodisiacs are associated with interactive factors located at the micro, meso, and macro levels of society (i.e., intrapersonal, interpersonal, community, organisational, and public policy factors).

### 4.1. Prevalence of Aphrodisiacs: Individual, Community, and Institutional Characteristics

The findings show that both men and women in Ghana commonly and frequently use aphrodisiacs. Participants attributed the proliferation and increasing use of aphrodisiacs to lapses in current regulations, community norms and practices, and individual behaviours. At the individual and community levels, the proliferation and increasing use of aphrodisiacs can be attributed to changing sexual awareness, curiosity, and overall behaviours among men and women. While the predominant cultural position on sexual relations is considered conservative, the prevalence of these drugs reflects an increased demand for them, as participants in this study asserted, and has been argued elsewhere [2,12]. Although aphrodisiacs were traditionally homemade using indigenous materials, businesses have capitalised on the socio-cultural premium placed on men’s sexual performance and female arousal to commercialise these products, making them readily available [2]. This position is supported by the recent work of Fiaveh [39], who demonstrates that even women, who have historically been considered sexually passive, are more active and not as passive as they were previously thought to be. As noted, “women have sexual agency pointing to the need to deconstruct misconceptions associated with female sexuality organised around masculine ideals” [39]. Thus, the prevalence of aphrodisiacs is, to a significant extent, a reflection of the changing societal discourse and attitudes towards sexual relations. However, the extent of this argument is limited in view of other findings in this study. For example, the role of social expectations, norms, and practices was apparent in terms of how they shaped women’s interest in and willingness to discuss sexual preferences and views, even with their partners, for fear of being stigmatised as sexually decadent [28]. While previous studies show that notions of women being passive in sexual relations are changing [39], such changes have been gradual; nonetheless, they have taken a fundamental turn. The fear of being stigmatised results in fewer opportunities for women to learn about sexual issues as they often censor their curiosity about the subject, at least in public, which leads to inadequate knowledge about sexual health matters [40,41].

Although individualised practices and decisions to engage with sexual enhancement drugs remain personal, the extent of such choices depends, to some degree, on the prevailing public policies relating to food and drug production, importation, and advertising. The significance of public policies in regulating the availability and use of sexual drugs is demonstrated by calls for governments to ensure the safety of available therapies proactively [1]. As evidenced by the findings of this study, the growing availability of approved and unapproved aphrodisiacs transcends the borders of Ghana. It is part of a global market, and so must its control be. Therefore, curbing the importation and advertisement of unapproved drugs and exercising more control over the use of approved ones is a starting point for protecting the public from unhealthy drug use. A cross-national pharmacovigilance collaboration programme is required to identify and prevent adulterated products [1]. These measures are critical considering the public and social policy implications of inaction in the long run, such as the inevitable rise in chronic diseases, which could burden the health system and social safety net provisions. In view of this study, agencies such as the Ghana Food and Drug Authority, which is mandated to regulate the safety and importation of food and drugs in the country, must strengthen its resolve in partnership with other agencies (e.g., Ghana Health Services, the media, and Customs Excise and Preventive Services) to monitor the production, distribution, and use of sexual enhancement drugs. Extant evidence suggests that there are lapses in current policy implementations (e.g., potential sale of approved drugs to underaged persons) [2]. In many ways, this suggestion is supported by calls in Europe [42] and the UK [43] to adopt a broad range of strategies to address threats posed by the misuse of aphrodisiacs.

### 4.2. Why Men and Women Use Aphrodisiacs: Multi-Level Perspectives

As revealed by this study, the reasons for using aphrodisiacs do not deviate markedly from those reported in existing studies in Ghana and globally across genders. The reasons include the stimulation of sexual urges, the desire to prolong intercourse, the wherewithal to initiate sex, the need to meet the expectations of partners, to increase the size of genitals (among men), and to enhance satisfaction for the users and their partners [2,11,44]. However, this study adds a multi-level perspective regarding the factors associated with the use of aphrodisiacs. This multi-level perspective revealed in this study indicates that aphrodisiac use is not determined by individual choices alone. It is also circumscribed by a web of intangible (e.g., religious beliefs and norms) and tangible (e.g., friends, partners) social factors beyond an individual’s control. The quantitative analyses showed that religiously inclined men were less likely to use aphrodisiacs. Considering that most religious faiths across Ghana abhor sexually “immoral” lifestyles [28], it is not surprising that these intangible virtues influence the behaviour of men. However, this finding and explanation somehow contravene the predominant cultural notion that expects men to be sexually strong to demonstrate their masculinity [2]. It will be interesting to examine in future research how men juggle between their religious faiths and the cultural/societal expectation of their sexual performance in the use of aphrodisiacs.

Consistent with the intrapersonal and community aspects of the social–ecological model, women were found to use aphrodisiacs because of perceived benefits to their partners. While such practices and attitudes may be the desire of some women, they are also likely to experience the consequence of long-held notions of patriarchal and legal systems that often dominate women’s sexualities and reproductive issues: “the capacity of African women to control their sexual and reproductive lives and to break free from the chains of domesticity is continually curtailed by law, culture and religion” [28]. This study observed that women who consumed alcohol regularly were more likely to use aphrodisiacs than those who seldom or never consumed alcoholic beverages. As many aphrodisiacs (including those approved and unapproved) are in the form of alcohol [20], it is understandable that people who consume alcoholic drinks are also likely to use substances that contain aphrodisiac ingredients. Evidence suggests that men tend to use substances such as alcohol more than women in Ghana [45]. This means that the likelihood of women who already engage in experimental drinking to take aphrodisiac-based drinks is high.

However, women who were employed were less likely to use aphrodisiacs than those who were unemployed. Unemployed women primarily include young adults, who are less likely to be in steady or long-term relationships that may encourage them to try aphrodisiacs. Indeed, young women are less likely to take drugs such as alcohol because of prohibitive religious and socio-cultural issues [45]. An alternative explanation to this finding is that employed persons may engage less in recreational sex, which may involve aphrodisiacs, than those employed because of the burden of household and professional work demands [46]. However, studies in the US show that both men and women maintain frequent sexual activities despite work [46]. Future studies could usefully find out more about the relationship between employment status and the use of aphrodisiacs using qualitative methods to provide a more detailed understanding of the use of aphrodisiacs.

A common determinant for both men and women regarding aphrodisiac use from the quantitative analysis was that those with multiple sexual partners were more likely to use aphrodisiacs than those with fewer or single partners. This finding is not surprising considering the recreational “fun” use of aphrodisiacs [2]. People who have multiple sexual partners may well use these drugs (whether approved or unapproved) to enable them to satisfy their numerous partners adequately, without feeling exerted, and to sustain those relationships, as reported elsewhere among men [9]. This study adds that such behaviours are not exclusive to men as they also occur among women.

### 4.3. Concerns about Health, Health Knowledge, and Aphrodisiac Use

The decision to use and the type of aphrodisiacs that people used were connected to participants’ understanding and knowledge of the effect of aphrodisiac use on their health. While studies in Ghana and other sub-Saharan African countries have shown the benefits of several aphrodisiacs in battling sexual problems such as erectile dysfunction [6], many participants perceived negative health outcomes in using these drugs in the short and long term. They based these negative perceptions on their personal and secondary experiences. Specifically, they intimated that the use of aphrodisiacs could cause chronic diseases and even death. From the study, and as observed in places such as Nigeria [11], this situation arises mainly because users of these drugs use unapproved and unprescribed ones. Such behaviours increase the likelihood of misuse (e.g., not following recommended dosage), which can result in poor health outcomes. Ease of access to these drugs, coupled with personal motivation—often underpinned by societal expectations, as discussed earlier—mean that people are less likely to adhere to drug precautions, if any. Thus, it is not a coincidence that participants, especially women, did not hold positive views about these drugs despite the prevalence of aphrodisiacs on the market and their widespread use. As we argue later, these perceptions highlight a need for improved public education, particularly on the variety of these drugs and how they work, at least for the approved ones. There appears to be a general perception that all the aphrodisiacs have deleterious health consequences without consideration for their evidence-based safety and benefits. This indicates a potential knowledge gap. Some men spoke favourably of aphrodisiac use from our findings by presenting it as a symbol of “love” if their partners proposed the drugs to them. This may explain why more men used these drugs than women, aside from the socio-cultural pressure to improve sexual performance. Therefore, the use of and attitudes toward aphrodisiacs have a strong interpersonal appeal that must be understood and integrated into public health educational activities. Moreover, public education on aphrodisiacs must adopt a relational approach whereby families, friends, and sex partners are urged to ensure responsible use of these drugs among their social networks.

Notwithstanding the public policy and health system weaknesses that encourage access to aphrodisiacs, negative health experiences can be attributed to intrapersonal factors such as inadequate health literacy among the adult population, especially women, as reported in other studies [47,48]. Health literacy describes a person’s ability to obtain, understand, and utilise information to make informed decisions about health [47]. It is evident that the use of unapproved aphrodisiacs and the general unadvised use of these drugs can be associated with the inability of both men and women to make informed health decisions. There is little doubt that organisational factors (i.e., the health system) and public policy factors also play a role in the public’s health literacy concerning the use of aphrodisiacs. Specifically, health promotion efforts need strengthening as regards access to proper information on each drug on the market (e.g., ingredients and side effects). This proposal has public health policy implications as it requires regulatory procedures to not only ensure the licensing of drugs but also ensure that the public has adequate information about each product. With these in mind, notions from participants that users of aphrodisiacs have less regard for their health are highly contestable as low health literacy is only a potential explanation for such actions. Future research should delve deeper into the gendered dimension of the relationship between health literacy and aphrodisiac use to identify ways to improve current sex education measures, as participants suggested.

In our study, participants attributed the need to use aphrodisiacs to poor health, which results from poor diets. Evidence suggests that poor health and chronic diseases are associated with the use of aphrodisiacs to overcome dissatisfaction with sexual performance. This is especially the case among men, as found in China [49]. Such a situation corresponds with claims by some participants in this study that men take to aphrodisiacs due to poor health and the growing incidence of sexual weakness. Among men in Ghana, the need to take aphrodisiacs arises from socio-cultural expectations to have high sexual stamina and performance [2], as “male sexual dysfunction in Ghana is acutely emasculating and embarrassing to men” and challenges traditional notions of what it means to be a man [50]. Such normative practices mean that men are likely to go to great lengths to meet these expectations, which is likely to be the case for those suffering from health problems.

In contrast, it was found that healthy women were more likely to use aphrodisiacs than unhealthy ones. Women are reluctant to engage in certain sexual conduct when they suspect poor environmental hygiene and personal health in either themselves or their spouses [20]. This indicates that favourable health-related conditions are critical for women to engage in sexual relations. Indeed, this study also found that women who engaged in physical activities (exercising) were less likely to use aphrodisiacs. Such women are likely to be more conscious of the health implications of taking medications for sexual purposes. Studies show that some Ghanaian women insist that their partners take measures to be physically fit as part of their sexual relations [20]. With these in mind, it is not improbable that such women are more likely to be cautious about engaging in habits that could negatively affect their health, including overly indulging in sexual enhancement drugs. Nonetheless, this explanation seems not to apply to women in this study. From the quantitative analyses, healthy women were more likely than those who were unhealthy to use aphrodisiacs. Given that aphrodisiacs are often taken for recreational purposes instead of diagnosed health problems, it is plausible that women who are in poor health are less likely to engage in medicinal therapies other than those taken for their ill-health. This is also because women may not face the same expectations as men to perform sexually.

## 5. Conclusions

This study has used qualitative and quantitative data to examine the multi-level factors that influence the use of aphrodisiacs among men and women in Ghana. The study found that different kinds of sexual enhancement drugs were commonly used, especially among men. Analysis of the differences and similarities between men and women provided evidence of individual characteristics (e.g., employment status, religiosity, number of sexual partners, exercising, and health status) associated with the use of aphrodisiacs from the survey analyses. The qualitative data indicated a critical role of community factors (e.g., social norms and expectations), interpersonal factors (e.g., expectations of partners and friends), public policy (e.g., drug-related regulations), and organisational/institutional factors (e.g., health system arrangements about access and use of drugs) in the use of aphrodisiacs among men and women. Although many participants, particularly women, had used aphrodisiacs, many of them, particularly women, held unfavourable views about the drugs due to perceived and experienced negative health implications for themselves and their partners. We argue that such perceptions and experiences may be influenced by the prevalence of unapproved drugs and inadequate knowledge about the different categories of these drugs and how they work. Cross-national collaborations are needed to eliminate adulterated products to protect the public from undesirable outcomes, particularly among men, as they use aphrodisiacs more frequently than women.

Altogether, these findings and the ensuing discussions reiterate the importance of understanding the use of sexual enhancement drugs among men and women from a multi-level perspective. Efforts to educate the public and regulate the production and use of these drugs should involve individual experiences, prevailing social norms, and a review of the health system arrangements and policy guidelines on these drugs. Such multi-level effort can help to improve public knowledge of these drugs and reduce the proliferation and utilisation of unsafe drugs. In the long term, such an approach will contribute to improving the health and well-being of the population.

### Limitations

This study used a mixed-method approach to examine the multi-level factors that influence the use of aphrodisiacs among men and women in Ghana. While this study expands on the current knowledge of the topic, some precautions must be observed in the interpretation and application of the findings. First, the study used cross-sectional data, which means that the findings only depict how participants felt at one point in time. Because of this, causal inferences cannot be drawn from the findings. The survey data did not include information about the characteristics of the aphrodisiacs that participants chose to use (e.g., whether they used approved or unapproved drugs, locally manufactured herbal drugs, or western medicines). Controlling these factors may have yielded different results. However, our qualitative data have addressed some of these limitations by offering insight into why people use these drugs. This justifies the confidence in the overall findings. Indeed, the findings are consistent with and add new perspectives to several existing research works. The study thus contributes to the ongoing discourse on promoting healthy sexual behaviours from multi-level and gender perspectives.

## Figures and Tables

**Table 2:** Factors associated with the use of aphrodisiacs by ordinal logistic regression ^#^.

	Men			Women			Overall		
	Estimate	Std. Error	95% Confidence Interval	Estimate	Std. Error	95% Confidence Interval	Estimate	Std. Error	95% Confidence Interval
**Sex**									
Male	--	--	--	--	--	--	1.343 ***	0.287	0.781, 1.905
Female	--	--	--	--	--	--			
**Religiosity**	−0.238 *	0.116	−0.465, −0.011	−0.207	0.290	−0.776, 0.362	−0.269 **	0.102	−0.469, −0.069
**Employment status**									
Employed	−1.705	0.653	−2.985, −0.425	−1.539 **	0.592	−2.700, −0.379	--	--	--
Pensioner	−11.005	694.788	−1372.764, 1350.755	−17.400	8891.899	−17,445.203, 17,410.402	--	--	--
Unemployed (ref)									
**Perceived health status**	0.225	0.138	−0.046, 0.496	0.869 *	0.379	0.126, 1.612	0.132	0.123	−0.109, 0.372
**Number of sexual partners**	0.481 **	0.170	0.148, 0.814	1.191 **	0.399	0.409, 1.974	0.757 ***	0.140	0.484, 1.031
**Alcohol intake**	0.203	0.171	−0.131, 0.538	1.041 **	0.372	0.311, 1.771	0.192	0.142	−0.087, 0.471
**Smoking**	0.109	0.152	−0.189, 0.407				−0.006	0.137	−0.275, 0.263
**Physical exercise**	--	--	--	−0.658 *	0.259	−1.167, −0.150	--	--	--
**Use of condom during last sexual encounter**									
No	−0.567	0.301	−1.157, 0.024	−0.529	0.658	−1.818, 0.760	−0.448	0.257	−0.952, 0.056
Yes (ref)									
*Nagelkerke R* ^2^	0.428			0.391			0.310		

^#^ All models were controlled for involvement in sexual intercourse, marital status, rural or uban residence, and region of residence. -- variable omitted due to lack of significant Spearman correlation. * *p* < 0.05, ** *p* < 0.01, *** *p* < 0.001.

**Table 3:** Characteristics of participants in the qualitative study.

Characteristic	Frequency (*n* = 31)	Percentage
**Age**		
18–35	14	45.2
36–49	11	35.5
50+	6	19.4
**Sex**		
Male	18	58.1
Female	13	41.9
**Educational attainment**		
Never been to school	2	6.5
Primary school	8	25.8
Junior high school/MLSC	14	45.2
SHS/A Level/O Level	4	12.9
Tertiary (including postgraduate)	3	9.6
**Religious affiliation**		
Christianity	16	51.6
Muslim	10	32.3
Traditionalist	1	3.3
No religion	4	12.9
**Marital status**		
Married	14	45.2
Not married	17	54.8
**Regions**		
Ashanti	18	58.1
Brong Ahafo	6	19.4
Greater Accra	7	22.6
**Area of residence**		
Urban	19	61.3
Rural	12	38.7

## Data Availability

The dataset analysed in this study is available from the corresponding author. Requests will be considered on a case-by-case basis.
